# HOW INTERNATIONAL EXPERTS DEFINE ADVANCE CARE PLANNING: A CONTENT ANALYSIS

**DOI:** 10.1093/geroni/igae098.0820

**Published:** 2024-12-31

**Authors:** Jenny van der Steen, Emma de Wit, Mandy Visser, Miharu Nakanishi, Lieve Van den Block, Ida Korfage, Jürgen In Der Schmitten, Rebecca Sudore

**Affiliations:** Leiden University Medical Center, Leiden, Zuid-Holland, Netherlands; Leiden University Medical Center, Leiden, Zuid-Holland, Netherlands; Topaz, Leiden, Zuid-Holland, Netherlands; Tohoku University Graduate School of Medicine, Sendai-shi, Miyagi, Japan; Vrije Universiteit Brussel (VUB), Brussels, Brussels Hoofdstedelijk Gewest, Belgium; Erasmus MC, University Medical Center, Rotterdam, Zuid-Holland, Netherlands; University of Duisburg-Essen, Essen, Nordrhein-Westfalen, Germany; University of California San Francisco, San Francisco, California, United States

## Abstract

Planning for future medical care, referred to as Advance Care Planning (ACP), has evolved to a focus on conversations that explore values and preferences in a broad sense. It is unclear how this conceptualization has been adopted internationally, given diverse practices. Therefore, we examined how experts from across the globe define ACP and whether this differs by profession. In an explorative study embedded in a Delphi study on ACP in dementia, experts in ACP in persons with dementia and other diseases reported at baseline how they define ACP “in one sentence, off the top of your head.” We analyzed reported definitions with content analysis, created codes to identify definition elements, then merged them into categories. Almost half (45%) of 87 experts from 30 countries phrased ACP from a patient perspective (29% neutral, 26% professional). Codes (131) were merged into 19 categories. Five categories appeared in more than half of the definitions: ‘Choosing between options’, ‘Care and treatment’, ‘Planning for the future’, ‘Individual person’ and ‘Having conversations.’ ‘End of life’ and ‘Documenting’ were mentioned by a minority of experts. The definition of categories and perspectives did not appreciably differ between physicians and other professions. In conclusion, international experts from 30 countries typically defined ACP as person-centered conversations to choose future care and treatment, without focusing on end of life or documentation. Future research should evaluate the extent to which such conceptualization of ACP is present within clinical programs and practice recommendations and monitor changes over time.

